# How local prevention efforts and state regulations intersect to reduce fatal shootings: a configurational analysis of firearm policies in the United States

**DOI:** 10.1057/s41271-026-00654-5

**Published:** 2026-08-03

**Authors:** Daniel C. Semenza, Edward J. Miech, Brielle Savage, Devon Ziminski

**Affiliations:** 1 Department of Urban-Global Public Health, Rutgers University, Piscataway, NJ USA; 2 Indiana University School of Medicine, Indianapolis IN, USA; 3 New Jersey Gun Violence Research Center, Piscataway, NJ USA; 4 School of Criminal Justice, Rutgers University, Newark, NJ USA; 5 Department of Health Sciences, Rutgers University, Camden, NJ USA

**Keywords:** Firearm violence prevention, Gun policy, Coincidence analysis (CNA), Urban firearm violence, Public health policy

## Abstract

Firearm violence arises from a complex interplay of local conditions and state regulations. Using coincidence analysis (CNA), we examined how combinations of city-level prevention efforts and state-level firearm policies correspond to fatal shooting rates across 100 US cities. Drawing on data from the Community Justice Violence Prevention Index, Everytown’s gun-law rankings, the US Census Bureau, and the Gun Violence Archive, we identified configurations of state policies and local efforts associated with relatively low rates of firearm-related fatalities. Five distinct pathways characterized cities with lower shooting rates, involving combinations of regional location, restraining order prohibitors, authority to deny firearm purchases, police use-of-force data collection, microstamping for new handguns, city population, ban-the-box initiatives, city-wide reporting for hate crimes, school-based violence prevention programs, and local emergency response improvements. Findings underscore how multilevel policy environments jointly shape firearm violence outcomes and demonstrate the value of configurational methods for public health and policy research.

**Supplementary Information:**

The online version contains supplementary material available at 10.1057/s41271-026-00654-5.

## Key

- Firearm violence is shaped by combinations of policies and local conditions. Multiple distinct configurations of state firearm regulations and city-level prevention efforts are linked to lower fatal shooting rates across U.S. cities.
- Configurational methods reveal complex pathways to lower rates of fatal shootings at the intersection of state laws and local interventions, offering a useful tool for public health and policy research.

## Introduction

Firearm violence in the United States (US) arises from a complex interplay of structural conditions, legal policies, and regional dynamics that vary across local communities, cities, and states [1,2]. A large body of research has analyzed how social conditions related to poverty, unemployment, structural racism, and concentrated disadvantage give rise to higher rates of firearm violence [3–6]. Scholars have similarly focused their attention on assessing the effectiveness of individual state-level policies that regulate ownership, sales, and legal use of firearms for reducing statewide rates of violence [7,8], while others have examined the efficacy of localized community-, hospital-, and police-based prevention strategies [9–12]. Despite this important work, few studies have examined how *combinations* of state policies and local strategies together influence rates of violence.

Although multiple, interwoven factors contribute to rates of firearm violence, researchers have rarely leveraged configurational methods like coincidence analysis (CNA) to assess how individual policies and strategies intersect to shape firearm violence outcomes. CNA is a cross-case, comparative method that relies on Boolean algebra to identify difference-making conditions for a particular outcome [13,14]. This method recognizes that interventions in public health, implementation science, and public policy typically involve inter-related elements that operate across multiple levels of the social ecology, enabling the identification of unique sets of conditions associated with a given outcome. In recent years, CNA has been increasingly used to assess combinations of factors for diverse outcomes, including vaccination uptake [15], school-based interventions for asthma self-management [16], nursing patient assignment [17], tumor screening [18], and cancer risk management [19].

Despite the growing popularity of CNA, only one study to date has used the method to assess firearm-related outcomes. Rich et al. (2016) used CNA to evaluate how combinations of ten different state-level firearm laws were linked to low rates of firearm suicide and homicide [20]. The authors found that three “pathways” comprised the minimum set of necessary and sufficient conditions for low state-level suicide rates. Policies within these pathways included the presence of universal background checks, the presence of minimum age requirements, and the presence of junk gun bans. In the same study, a three-pathway model for low homicide rates included firearm policies, such as the presence of minimum age restrictions and the presence of background checks. The findings underscore the importance of considering policies and other contextual factors in conjunction with one another when developing holistic approaches to firearm violence prevention.

Although the study by Rich et al. represents an important first investigation of firearm violence rates using CNA, the paper focused exclusively on state-level policies without accounting for localized, city-level strategies. The present study expands this work by integrating novel data on local city policies to address firearm violence with data on state-level firearm policies across 100 US cities. We use CNA to analyze how combinations of both local- and state-level policies correspond to low rates of fatal shootings. The results provide insight into how prevention efforts at intersecting levels of the social ecology influence local firearm fatality rates while highlighting the use of configurational comparative methods like CNA for ongoing firearm violence prevention research.

## Data and methods

We collected data from multiple sources to conduct the current study. All data sources are from 2023. First, we used data from the Community Justice Violence Prevention Index (VPI) [21], which includes 34 indicators of city-level violence prevention efforts based on a public health framework for the 100 cities that experienced the highest rates of gun violence, based on data from the Gun Violence Archive. Community Justice, a non-profit violence prevention organization, created individual “score cards” for each city to determine the city’s readiness for community firearm violence prevention, scoring the city based on the absence or presence of local policies in 2023 across the following ten areas: immediate intervention, risk factor reduction, housing and food insecurity, employment, education, health, youth and families, firearm laws and regulations, crime reporting and data, and offices of violence prevention.

VPI scorecards were generated by Community Justice Staff using publicly available information and then validated by contact with city representatives through mayors’ offices, city managers’ officers, and city offices of violence prevention. City representatives were given at least 6 weeks to email requested changes for all items included on the scorecards and any supporting documentation. Members of the Community Justice VPI team reviewed all requests and documentation received within the feedback period to produce the final scorecard for each city. Scorecards were downloaded and each city was coded for the absence (0) or presence (1) of the 34 individual policies across the 10 main policy areas. Please see Appendix A for a full list of the 100 cities included and Appendix B for all 34 indicators included in the index.

Second, we collected 2023 fatal city-level shooting data for each of the 100 cities included in the VPI from the Gun Violence Archive, a publicly available epidemiologic resource for measuring firearm violence [22]. We used city-level population estimates from the American Census Bureau for 2023 to calculate rates of fatal shootings for each city. The shooting rates do not include suicides. To calculate the fatal shooting rate we divided the number of fatal shootings for 2023 for each city by the total population of the city. We then multiplied each rate by 10^5^ to calculate an average shooting rate per 100,000 people.

Since our analytic approach required our outcome to be expressed as a categorical factor, we calculated tertiles for the outcome of fatal shooting rates and divided the 100 cities into two groups. The 33 cities that fell within the lowest tertile for fatal shooting rates were assigned an outcome value of “1,” whereas the other 67 cities were assigned an outcome value of “0.” This approach allowed us to identify configurations of policies and contextual conditions directly linked with lower levels of fatal shootings while maintaining sufficient variation across cases for configurational modeling.

Third, we identified the presence or absence of 50 individual state-level firearm policies across all 50 states and Washington DC using data from Everytown’s 2023 gun-law rankings (https://everytownresearch.org/rankings/). Although there are numerous sources available for measuring state-level firearm policies, we used these indicators from Everytown, since they are publicly accessible, were updated for 2023 at the time of analysis, and have been validated alongside other indices of state-level policies [23]. We coded for the absence (0) or presence (1) of each of the state-level policies in the 100 cities in our database. See Appendix C for a full list of state-level policies included in the dataset.

Two additional measures were included as contextual factors in the CNA models. City population was categorized as follows: > 1 million people was assigned a “4”; 600,000 to 1 million people was assigned a “3”; 300,000–600,000 people was assigned a “2”; and < 300,000 people was assigned a “1.” We included the regional division of the country for each city/state based on the nine designations used by the US Census Bureau (Northeast: New England, or Middle Atlantic; South: South Atlantic, East South Central, or West South Central; Midwest: East North Central, Midwest or West North Central; West Mountain, or Pacific) given prior research that accounts for regionality when assessing firearm outcomes using CNA [20, 24].

### Analytic strategy

Coincidence analysis was used to identify difference-making conditions accounting for lower fatal shooting rates at the city level. CNA is a case-based approach to data analysis that uses Boolean algebra, set theory, and formal logic to determine crucial conditions that consistently and uniquely yield an outcome of interest [14, 25–27]. CNA is designed to embrace real-world complexity and researchers using CNA can identify difference-making conjuncts and disjuncts to assess what makes a difference, for whom, and under what conditions. Conjuncts represent bundles of conditions that jointly appear together and draw upon the Boolean operator “AND.” Disjuncts occur when there are multiple pathways that lead to the same outcome and operationalize the Boolean operator “OR.” CNA can be applied to small and large sample sizes and yields different sets of findings that can complement results generated through standard regression-based methods [28,29]. The R package “cna”, RStudio, and Microsoft Excel were used for the analysis [30].

Please see Appendix D for the full methodological details of all CNA phases carried out for the current analysis. To assess whether our model was robust across alternative definitions of the outcome, we conducted two sensitivity analyses using the lowest quartile for fatal shootings (the 25 cities in our dataset with the lowest fatal shooting rates) and a second cutoff below the median (the 50 cities with the lowest fatal shooting rates).

## Results

Fatal shooting rates varied from a minimum of 1.95 shootings per 100,000 people for San Diego, California to a maximum of 164.43 shootings per 100,000 people for Decatur, Georgia. Thirty-three cities met the cutoff value of less than 12.20 shootings per 100,000 people to belong in the lowest tertile of total shootings. As a note for interpreting the figures, cells with yellow highlighter both have the outcome present and are covered by the model (consistent cases), whereas cells with green highlighter are covered by the model but do not have the outcome present (inconsistent cases).

The exploratory data analysis identified eleven factors to carry forward into the modeling phase: region, having an emergency restraining order prohibitor policy (state), having the authority to deny firearm purchases for public safety (state), police use-of-force data collection (state), microstamping for new handguns (state), population (city), municipal ban-the-box initiatives (city), data collection and reporting for hate crimes (city), school-based violence prevention programs (city), comprehensive city-wide plans for addressing violence (city), and having local efforts to improve EMS quality and response times in impacted communities (city).

**Fig. 1:**
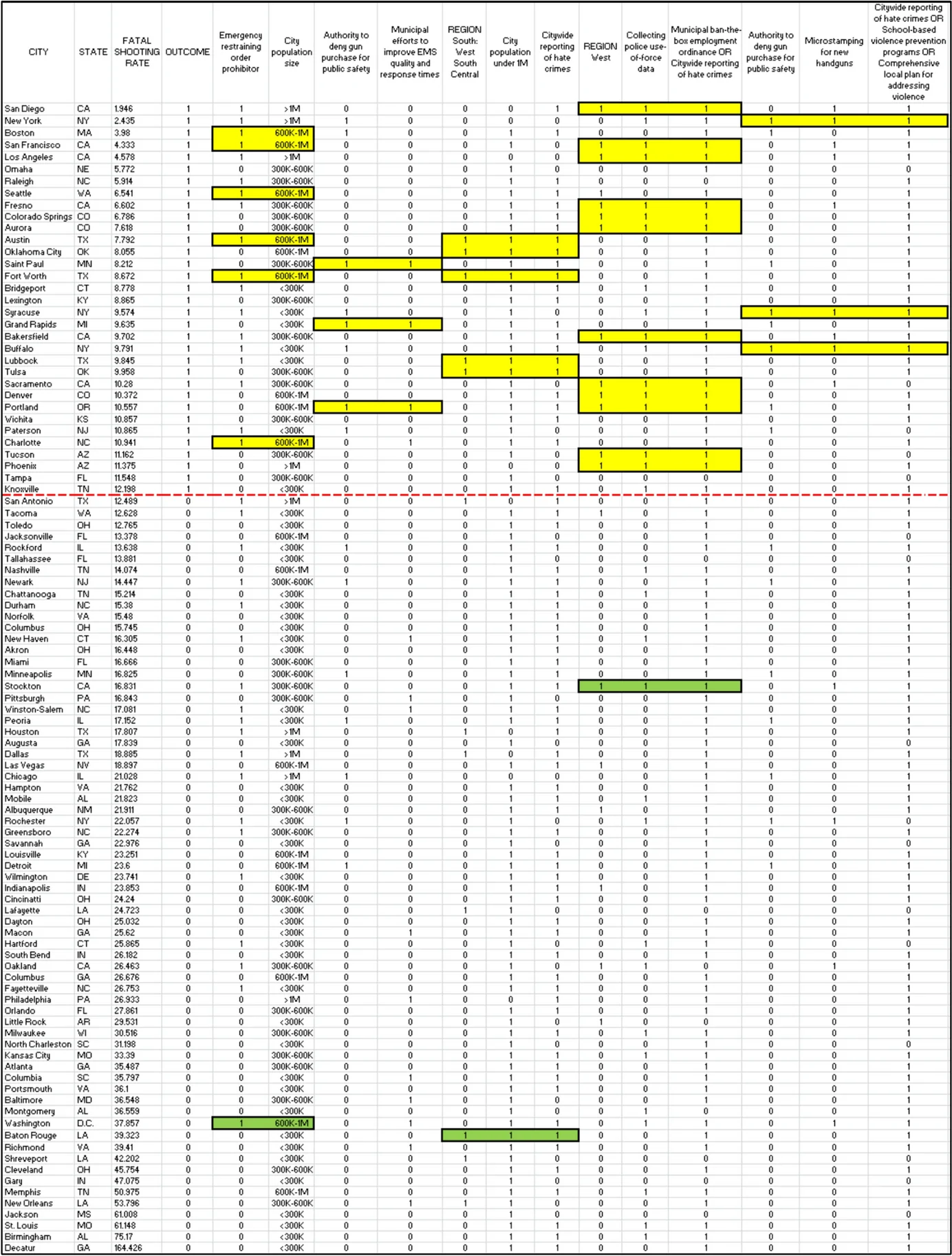
CNA model for 33 cities with lowest rates of fatal shootings. *Notes* The dotted red line separates the 33 cities with the outcome of lower total shooting rate (above the dotted red line) from those without (below the line). Cells with yellow highlighter both have the outcome present and are covered by the model (consistent cases), whereas cells with green highlighter are covered by the model but do not have the outcome present (inconsistent cases). Thick boundary lines indicate conjunctions, where two conditions must be jointly present to yield the outcome

The modeling phase identified a five-pathway model that was highly reliable with an overall consistency score of 89.3% (25 of the 28 cities identified by the model were in the lowest tertile) and accounted for 25 of the 33 cities in the lowest tertile for a standard coverage score of 75.8% (28/33). The prevalence-adjusted measures for consistency (94.4%) and contrapositive coverage (79.8%) were even higher. Figure 1 shows each of the five pathways and the cities they cover.

The five pathways found include: (Pathway A) having a population between 600,000 and 1 million (city) AND having an emergency restraining order prohibitor in place (state); (Pathway B) having the authority to deny gun purchase for public safety (state) AND having local efforts to improve EMS quality and response times in impacted communities (city); (Pathway C) being in region 7 (South: West South Central region) AND having a population of less than 1 million (city) AND collecting and reporting hate crime data (city); (Pathway D) being in region 8 or 9 (West: Mountain or Pacific) AND police use-of-force data collection (state) AND at least one of the following two local prevention measures: collecting and reporting hate crime data (city) or a "ban the box" policy (city); and (Pathway E) having the authority to deny gun purchases for public safety (state) AND microstamping of new handguns (state) AND at least one of the following three local prevention measures: collecting and reporting hate crime data (city), school-based violence prevention programs (city), or a comprehensive city-wide plan for addressing violence (city).

The sensitivity analysis using the lowest quartile as an alternative threshold (the 25 cities in the dataset with the lowest fatal shooting rates) showed that this model maintained a high level of reliability with a consistency score of 75% (prevalence-adjusted consistency = 90%, coverage = 84%, prevalence-adjusted contrapositive coverage = 85%). In the second sensitivity analysis that used a below-the-median cutoff (the 50 cities in the dataset with the lowest fatal shooting rates), the model likewise demonstrated high reliability with a consistency score of 92.9%, still accounting for the majority of cities with the outcome (coverage score = 52%). The prevalence-adjusted score for consistency was identical to standard consistency given the 50/50 balance in the distribution of the outcome, and prevalence-adjusted contrapositive coverage was higher than standard coverage at 66.7%. Appendix E and F show detailed figures for the model when using these two alternative definitions of the outcome.

## Discussion

This study examined how configurations of local- and state-level policies link to fatal shooting rates across 100 US cities. Our use of CNA methods illustrates the interplay of local firearm policies with state-level regulations and demographic considerations to complement findings generated through standard quantitative approaches that identify individual risk factors for firearm-violence-related outcomes. Rather than examine the association between a single independent variable and a dependent variable, our CNA analysis identified difference-making bundles of conditions that were necessary and sufficient for the outcome of interest (low rates of fatal shootings). Starting with a large initial set of nearly 90 potential explanatory factors (50 state-level firearm policies, 34 city-level violence prevention efforts/policies, population, and region), the CNA models pinpointed five pathways that consistently and uniquely distinguished cities with comparably low fatal shooting rates.

Prior research has supported a reduction in violence associated with restrictions against those prohibited from owning a firearm [31]. Pathway A (having a population between 600,000 and 1 million AND having an emergency restraining order prohibitor in place (state) and Pathway B (having the authority to deny firearm purchase for public safety (state) AND having local efforts to improve EMS quality and response times in impacted communities (city) both highlight how state-level policies—firearm restrictions for those with a restraining order and denial of firearm purchases—can combine with population and city-level resource considerations (such as improved EMS services) to influence local firearm violence rates. Pathway A notably suggests that protective policies could operate most effectively in large cities. Pathway B findings may be interpreted such that the availability of firearm purchase denials and improved emergency services contribute to better overall public safety and more robust city-wide prevention efforts, thus shaping local contexts into places where shootings are less likely to occur.

Each pathway identified in our results is a conjunction of discrete conditions. Notably, our results do not point to a single, clear-cut pathway toward lower rates of fatal shootings. The interplay of each conjunct highlights the challenge of policy effectiveness in complex environments. Identifying and isolating the effects from one policy can be difficult, and desired outcomes are likely influenced by a combination of many different policies, programs, and related contextual factors, such as demographics and regional cultures as well as implementation effectiveness. Relatedly, the history of any given city’s policy efforts also matters. For instance, a city with a long history of local prevention efforts may be affected differently by new state regulations than a city with limited local prevention efforts (and vice versa). Yet it is notable that certain policies spanned multiple pathways in the model, including policies to prevent potentially dangerous people from accessing firearms (state) and collecting and reporting hate crime data (city).

The finding that the EMS response measure is a difference maker as part of Pathway B is particularly interesting, because it entails efforts that include the use of supplemental county EMS services to support city EMS services, utilization of city firefighters as supplemental EMS responders, incorporation of strong and clear quality standards and audits into EMS provider contracts, and the use of data and technology to improve logistics [21]. There may be variation in how services are improved across cities, but better supported EMS in general may translate into higher quality care. Ultimately, regulations are implemented and enforced in hyperlocal contexts, so uncovering an ecosystem of inter-related policies can help identify the most comprehensive and effective firearm-policy bundles available for adequate prevention [32]. The regional factors’ presence in the model also highlights how geography in a large country like the US influences conditions for firearm-policy uptake and effectiveness [33].

This study is not without limitations. First, the data included in the analysis are cross-sectional, representing a single year of information. The firearm policies included in the analysis represent provisions in force in 2023, rather than newly enacted laws from that year. As such, the policy variables capture the broader policy environment within which cities operate. Although many of the state-level firearm policies included in the analysis represent longstanding legal provisions, we do not observe policy enactment dates or duration of exposure, and the timing of local prevention efforts likely varies across cities. As a result, the identified configurations should be interpreted as links between policy environments and firearm violence rather than definitive causal effects. Some local interventions such as EMS improvements may be implemented in response to elevated violence, raising the possibility of reverse causation. Longitudinal and quasi-experimental research will be important for assessing how changes in policy environments over time influence firearm violence outcomes.

Second, while the model had high consistency and coverage scores, there were nonetheless eight cities with the outcome present that remained unaccounted for, indicating a role for additional factors beyond those included in our dataset. Firearm ownership, socioeconomic conditions, law enforcement strategies, demographic characteristics, and recent events such as the COVID-19 pandemic all likely contribute to shaping the local context of fatal shootings but were not included in the present analysis. While incorporating such environmental factors could provide additional insight into firearm violence patterns, we were cautious in bracketing our CNA analysis to focus on policy-relevant combinations of state-level policies and city-level prevention measures. We encourage future CNA researchers to consider these local contextual factors in future analyses, as well as assessing configurations of law enforcement strategies such as focused deterrence, specialized anti-gun violence task forces, citywide CCTV systems, acoustic gunshot detection technologies, and variations in departmental budgets or operational priorities alongside local prevention efforts and state firearm policies.

Third, factors not included in our model should not automatically be considered unimportant or irrelevant for the main outcome. Rather, the results indicate these factors do not meet the definition of difference makers within CNA (the minimum set of necessary and sufficient conditions for the outcome to appear) but may still play other important roles in violence reduction. Future research that includes information prior to the COVID-19 pandemic as well as more recent data after 2023 will be important for assessing whether the configurations identified here persist as policy environments and social conditions evolve.

Finally, our data are limited to measuring the presence of policies and we did not have information about the fidelity or intensity of local prevention efforts or state policy implementation. Variation in implementation efficacy likely plays a significant role in predicting policy success and helps account for some of the patterns in our data. To the extent possible, we encourage researchers to consider implementation fidelity in future CNA work. Relatedly, our analysis is limited to the use of the VPI scorecard measures, created using publicly available data and feedback from individual city representatives. As noted in the 2023 Community Justice report [21], it remains possible that scores may not fully reflect violence prevention efforts if public information was outdated and city respondents did not respond to requests for feedback.

## Conclusions

This study advances our understanding of how local- and state-level policies combine to shape local environments more or less conducive to firearm violence. The CNA approach offers a promising avenue for advancing the field’s assessment of conjunctive firearm policies, underscoring the need to complement traditional quantitative methods of identifying the effects of single policies using standard regression models. We encourage researchers to prioritize understanding how policies combine with one another at multiple levels of the social ecology and within different geodemographic contexts. Beyond policy assessment, firearm researchers may consider using CNA to evaluate how particular components of community violence interventions, focused policing approaches, hospital-based programs, and other prevention initiatives combine with one another to produce the most effective outcomes. This analytic approach offers an important implement within the broader methodological toolkit for advancing knowledge toward continued reductions in firearm violence across the US.

## Supplementary Information

Below is the link to the electronic supplementary material mentioned as Apendices in the text.

Supplementary file1 (DOCX 205 kb)

## Data Availability

Data will be made available upon reasonable request from the corresponding author.
