# Supplementary material for: How local prevention efforts and state regulations intersect to reduce fatal shootings: a configurational analysis of firearm policies in the United States
Source: J Public Health Policy. 2026 Aug 3;47(3):387–98. doi: 10.1057/s41271-026-00654-5 (PMC13489947; doi:10.1057/s41271-026-00654-5)
Supplement: Supplementary file 1 [file 41271_2026_654_MOESM1_ESM.docx]

**Appendix**

**Appendix A.** VPI cities

2. Akron
3. Albuquerque
4. Atlanta
5. Augusta
6. Aurora
7. Austin
8. Bakersfield
9. Baltimore
10. Baton Rouge
11. Birmingham
12. Boston
13. Bridgeport
14. Buffalo
15. Charlotte
16. Chattanooga
17. Chicago
18. Cincinnati
19. Cleveland
20. Colorado Springs
21. Columbia
22. Columbus
23. Columbus
24. Dallas
25. Dayton
26. Decatur
27. Denver
28. Detroit
29. Durham
30. Fayetteville
31. Fort Worth
32. Fresno
33. Gary
34. Grand Rapids
35. Greensboro
36. Hampton
37. Hartford
38. Houston
39. Indianapolis
40. Jackson
41. Jacksonville
42. Kansas City
43. Knoxville
44. Lafayette
45. Las Vegas
46. Lexington
47. Little Rock
48. Los Angeles
49. Louisville
50. Lubbock
51. Macon
52. Memphis
53. Miami
54. Milwaukee
55. Minneapolis
56. Mobile
57. Montgomery
58. Nashville
59. New Haven
60. New Orleans
61. New York
62. Newark
63. Norfolk
64. North Charleston
65. Oakland
66. Oklahoma City
67. Omaha
68. Orlando
69. Paterson
70. Peoria
71. Philadelphia
72. Phoenix
73. Pittsburgh
74. Portland
75. Portsmouth
76. Raleigh
77. Richmond
78. Rochester
79. Rockford
80. Sacramento
81. Saint Paul
82. San Antonio
83. San Diego
84. San Francisco
85. Savannah
86. Seattle
87. Shreveport
88. South Bend
89. St. Louis
90. Stockton
91. Syracuse
92. Tacoma
93. Tallahassee
94. Tampa
95. Toledo
96. Tucson
97. Tulsa
98. Washington
99. Wichita
100. Wilmington
101. Winston-Salem

**Appendix B.** 2023 Violence Prevention Index Scorecard items in 100 cities

1. Intervention
   a. Outreach-Based Violence Intervention Programs
   b. Hospital-Based Violence Intervention Programs
   c. Cognitive Behavioral Therapy-Integrated Mentorship Programs for At-Risk Individuals
2. Risk-Factor Reduction
   a. Re-entry Programs and Services for At-Risk Returning Residents
   b. Municipal Diversion Programs
   c. Restorative Justice Programs
   d. Emergency and Transitional Housing
3. Housing and Food Insecurity
   a. Housing and Food Assistance Programs for Impacted Communities
4. Employment
   a. Strategic Workforce Development Programs
   b. Municipal Ban-the-Box Ordinances
5. Education
   a. Safe Passage Programs
   b. School-Based Violence Prevention Programs
   c. School Suicide Prevention Policies and Programs
   d. Inclusive School District Anti-Bullying Policies
6. Health
   a. Local Health Department Violence Prevention Strategy
   b. Trauma Recovery Centers
   c. Long-Term Trauma-Informed Behavioral & Mental Health Programs for Impacted Communities
   d. Community-Based Mental Health Crisis Response Programs
   e. Local Supplemental Violent Crime Victim Compensation Programs
   f. Municipal Strategies to Address Trauma Center Deserts in Impacted Communities
   g. Municipal Efforts to Improve EMS Quality and Response Times in Impacted Communities
7. Youth and Families
   a. Youth and Family Violence Prevention Programs and Survivor Services
   b. Intimate Partner Violence Prevention Programs
8. Local Firearm Laws and Regulations
   a. Ghost Gun Prevention Ordinances
   b. Background Check and Permit-to-Purchase Requirements
   c. Anti-Gun Trafficking Initiatives
9. Crime Reporting Data
   a. High Violent Crime Clearance Rates (above 70%)
   b. Hate Crimes Data Collection and Reporting
10. Local Office of Violence Prevention
    a. Established Local Office of Violence Prevention (OVP)
    b. OVP Comprehensive Plan for Addressing Violence
    c. OVP Pursuit of Grant Opportunities to Support Violence Prevention
    d. OVP Funds and Coordinates Violence Intervention Programming
    e. OVP Provides Technical Assistance
    f. OVP Oversees Evaluations of Local Programming

**Appendix C.** State firearm policies (Everytown Gun Law Rankings, 2023)

2. Background check and/or purchase permit
3. Concealed carry permit required
4. Extreme risk law
5. No shoot first law
6. Secure storage/child access prevention required
7. Assault weapons prohibited
8. Consumer safety
9. Ghost guns regulated
10. High capacity magazines prohibited
11. Microstamping for new handguns
12. Gun industry legal accountability
13. Crime gun tracing
14. No carry after violent offense
15. No guns - college
16. No guns - capitols
17. No guns - bars
18. No guns - K-12 schools
19. Open carry regulated
20. Strong concealed carry authority
21. Emergency restraining order prohibitor
22. Felony prohibitor
23. Fugitive from justice prohibitor
24. Gun removal program
25. Hate crime prohibitor
26. Mental health prohibitor
27. Minimum age to purchase
28. No gun purchase after violent offense
29. Prohibition for convicted domestic abuser
30. Prohibition for domestic abuser under restraining order
31. Relinquishment for convicted abusers
32. Relinquishment for abusers under restraining order
34. School threat assessment team
35. Stalker prohibitor
36. Funding services for victims of gun violence
37. Local gun laws allowed
38. No law enforcement officers bill of rights
39. Office of violence intervention
40. Police use of deadly force standard
41. Police use of force data collection
42. Qualified immunity limited
43. Violence intervention program funding
44. Authority to deny gun purchase for public safety
45. Charleston loophole closed or limited
46. Dealer license required
47. Lost and stolen reporting
48. Mental health record reporting
49. Notification of failed background checks
50. Sales records sent to law enforcement
51. Training required to purchase guns
52. Waiting periods

**Appendix D.** Methodological detail for coincidence analysis (CNA) analytic strategy

The first phase in the CNA analysis involved data reduction. To achieve this, an exploratory data analysis was conducted using the “minimally sufficient conditions” (*msc*) function, a routine that has been described in prior research [1-3]. The *msc* function was applied within the R package “cna” to look across all 100 cities and all 87 potential explanatory factors at once in the original dataset to identify combinations or “bundles” of conditions with especially strong connections to the primary outcome of interest: belonging to the lowest tertile for fatal shootings. This exhaustive process considered every possible 1-, 2-, and 3-condition combination of values within the dataset, retaining those bundles that met the specified consistency threshold. During this exploratory data analysis, the *msc* function was run at five different consistency levels: 95%, 90%, 85%, 80% and 75%.

We then analyzed the output generated by the *msc* routine to identify configurations with “best of class” coverage scores: bundles with top coverage scores for their complexity score (bundles with the same number of conditions) and with a substantially higher coverage score than its next-nearest neighbor (the bundle with the second-highest coverage score at the same complexity level). This *msc* routine identified candidate bundles with a particularly strong connection to the outcome of interest, which we then further vetted to ensure that they were consistent with logic, theory, and prior knowledge. For theoretical reasons, we focused on configurations with the presence of state policies or local prevention measures, as it was not expected that the absence of firearm policies or prevention measures would plausibly link to lower fatal shooting rates. We inductively analyzed the entire dataset for the outcomes of interest, gathered the subset of factors represented within these configurations, and carried them forward into model development during the next step of the CNA analysis.

Using the subset of factors selected during the exploratory data analysis, our goal during the modeling phase was to identify an overall model with ≥75% scores for both consistency and coverage; that was aligned with theoretical understanding and background knowledge; and where each pathway included at least one city-relevant condition. Consistency is a measure of reliability, indicating how often cases identified by the model also have the outcome present. Coverage is a measure of explanatory breadth, indicating how many cases that have the outcome present are covered by the model [4]. Because the outcome was imbalanced in our primary analysis (33 cities had the outcome of lower fatal shooting rates, whereas 67 did not), we report prevalence-adjusted consistency and coverage scores in addition to standard consistency and coverage scores [5].

**Appendix E.** Sensitivity analysis using the lowest quartile outcome definition (25 cities with the lowest rates of fatal shootings)

**Appendix F.** Sensitivity analysis using the below-the-median outcome definition (50 cities with the lowest rates of fatal shootings)

**References**

1. Roczniewska M, Tafvelin S, Nielsen K, et al. Simple roads to failure, complex paths to success: an evaluation of conditions explaining perceived fit of an organizational occupational health intervention. *Appl Psychol*. 2024;73(3):1103-1130.
2. Wasmuth S, Bravata D, Miech E. Difference-making conditions for the successful implementation of IDEAS: A provider stigma-reduction training for and by occupational therapy practitioners. *Am J Occup Ther*. 2025;79(5):7905205070.
3. Pratt-Chapman ML, Miech EJ, Mullins MA, et al. Difference-makers for collecting sexual orientation and gender identity data in oncology settings. *Cancer Med*. 2025;14(5):e70727.
4. Whitaker RG, Sperber N, Baumgartner M, et al. Coincidence analysis: a new method for causal inference in implementation science. *Implement Sci*. 2020;15:1-10.
5. De Souter L, Baumgartner M. New sufficiency and necessity measures for model building with Coincidence Analysis. Zenodo; 2025.
